# Potential Applications of Nanotechnology in Urological Cancer

**DOI:** 10.3389/fphar.2018.00745

**Published:** 2018-07-09

**Authors:** Ming-Hui He, Li Chen, Ting Zheng, Yu Tu, Qian He, Hua-Lin Fu, Ju-Chun Lin, Wei Zhang, Gang Shu, Lili He, Zhi-Xiang Yuan

**Affiliations:** ^1^Department of Pharmacy, College of Veterinary Medicine, Sichuan Agricultural University, Chengdu, China; ^2^College of Pharmacy, Southwest Minzu University, Chengdu, China

**Keywords:** nanotechnology, urological cancer, nanoparticles, diagnosis, therapy

## Abstract

Nowadays, the potential scope of nanotechnology in uro-oncology (cancers of the prostate, bladder, and kidney) is broad, ranging from drug delivery, prevention, and diagnosis to treatment. Novel drug delivery methods using magnetic nanoparticles, gold nanoparticles, and polymeric nanoparticles have been investigated in prostate cancer. Additionally, renal cancer treatment may be profoundly influenced by applications of nanotechnology principles. Various nanoparticle-based strategies for kidney cancer therapy have been proposed. Partly due to the dilution of drug concentrations by urine production, causing inadequate drug delivery to tumor cells in the treatment of bladder cancer, various multifunctional bladder-targeted nanoparticles have been developed to enhance therapeutic efficiency. In each of these cancer research fields, nanotechnology has shown several advantages over widely used traditional methods. Different types of nanoparticles improve the solubility of poorly soluble drugs, and multifunctional nanoparticles have good specificity toward prostate, renal, and bladder cancer. Moreover, nanotechnology can also combine with other novel technologies to further enhance effectivity. As our understanding of nanotechnologies grows, additional opportunities to improve the diagnosis and treatment of urological cancer are excepted to arise. In this review, we focus on nanotechnologies with potential applications in urological cancer therapy and highlight clinical areas that would benefit from nanoparticle therapy.

## Introduction

Urological cancers, which include cancers of the prostate, kidneys, and bladder, are an international public health problem. Minimally invasive surgical approaches are now the most common treatments for urological malignancies, although open radical nephrectomy was the most common treatment for non-metastasized lesions in the 1980s. Most prostate cancers, regardless of tumor size, are treated by laparoscopic nephrectomy (Rassweiler et al., 2013). In contrast, bladder cancer (BCa) is treated with different approaches depending on the stage of the cancer. Transurethral resection of bladder tumors (TUR-BT) is the first strategy for curing non-invasive bladder tumors, and Bacillus Cal-mette-Guérin (BCG) and chemotherapeutic agents are also widely used (Chen et al., 2015a). Many novel techniques for use in urological cancer have been developed in the past decade. For example, focal ablation using cryotherapy or radiofrequency (RF) is becoming more popular for small renal masses (Park et al., 2006; Lusch et al., 2013), and new methods, such as real-time peripheral temperature monitoring, are involved in establishing the efficacy of RF ablation (Leveillee et al., 2013). Similarly, in the treatment of prostate tumors, high-intensity focused ultrasound, a minimally invasive therapy, can be accurately targeted to a portion of the prostate gland and has been widely used for the treatment of prostate cancer (Crouzet et al., 2014). However, these traditional surgical therapies sometimes lack accuracy and have a high risk of complications, including tumor relapse (Kondylis et al., 2000). Moreover, the use of chemical agents may cause negative side effects in normal cells and could be associated with relapse.

Imaging of tumors can facilitate early diagnosis and influence patients’ treatment decisions. Computed tomography (CT), Positron emission tomography (PET), and magnetic resonance imaging (MRI) are traditionally used for detection of non-invasive malignancies. However, these traditional approaches have limited sensitivity and ability to provide specific and functional information on the disease (Wang et al., 2008).

**FIGURE 1:**
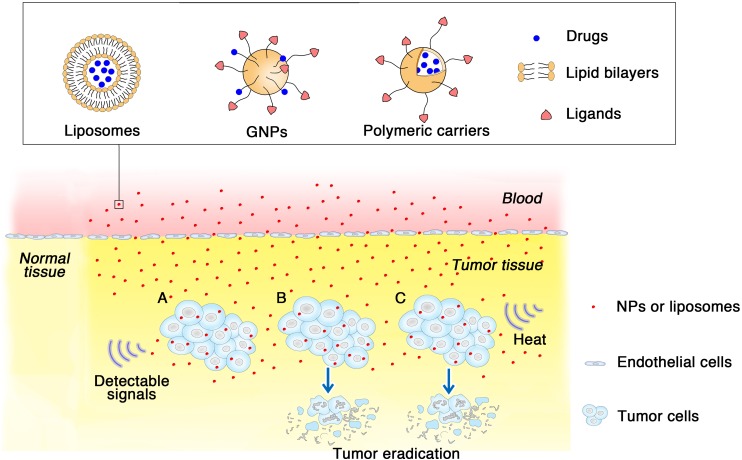
How different kinds of NPs applied in urological cancer diagnosis and therapeutics. Three types of NPs are listed for examples, liposomes, GNPs and polymeric NPs. With specific ligands modified on the surface, NPs show specifia targeting to the tumor cells via EPR effect. **(A)** NPs as imaging agents gather around tumor tissues then send and amplify detectable signals which are received subsequently. **(B)** NPs loaded with cytotoxic drugs arrive at tumor tissue, and then lead to tumor eradication. **(C)** In hyperthermia therapy, NPs absorbing heat energy induce a high temperature, causing focal cell death and preserve the circumambient normal tissue.

Nanoparticles (NPs, size in nanometer range) are novel materials used that were initially used for invention of the scanning probe microscopy and the discovery of molecular structures (Menter et al., 2014). Different manufacturing methods and materials have enabled the production of NPs with various shapes and sizes. One nanometer (10^-9^ m) is the scale at which most biological molecules operate inside living cells; thus, NPs may have many applications in the field of medicine. NPs can accumulate in tumor tissues via the enhanced permeability and retention (EPR) effect; under certain circumstances, typically for tumors, the endothelial lining of the blood vessel wall becomes more permeable than in the normal state, allowing NPs to enter tumors (Ojha et al., 2017). NPs can be used for encapsulation of poorly soluble drugs, protection of therapeutic molecules, modification of their blood circulation and tissue distribution profiles, and facilitation of combination regimens commonly used in cancer therapy (Bertrand et al., 2014). Many types of NPs, including liposomes and other lipid-based carriers (such as lipid emulsions and lipid-drug complexes), polymer-drug conjugates, polymer microspheres, micelles, and various ligand-targeted products (such as immunoconjugates), are under various stages of development as drug delivery systems (Wang et al., 2008). Additionally, NPs play an important role in cancer diagnosis, and may have applications in the treatment of tumors (**Figure 1**). Contemporary cancer therapy, particularly with respect to drug delivery, has evolved from traditional methodology to new technology. Thus, nanotechnology-based combination drug delivery to tumor tissues has emerged as a potential effective strategy for cancer therapy.

Nanotechnology has shown several advantages over widely used traditional methods. Different types of NPs improve the solubility of poorly soluble drugs and multifunctional NPs have good specificity toward prostate, renal and bladder cancers. Moreover, nanotechnology can also combine with other upcoming technologies and enhance the effectivity. As our understanding of nanotechnologies grows, opportunities to improve the diagnosis and treatment methods for urological cancer have also increased. In this review, we focus on nanotechnologies with potential applications in prostate, renal, bladder, and other urologic cancer. In each of these fields, we discuss various NP-based strategies for diagnosis as well as treatment. In addition, we provide future perspectives to highlight the requirement for further studies.

## Nanotechnology in Prostate Cancer

Prostate cancer is one of the leading causes of cancer-related deaths among men (Siegel et al., 2018). Chemotherapeutic agents, such as docetaxel and paclitaxel, which are widely used for the treatment of prostate cancer, are not selective to cancerous tissues and can cause injury to normal tissues, leading to a low therapeutic index and even increasing drug resistance. Consequently, novel approaches are urgently needed for the development of improved therapeutic options for the management of prostate cancer, particularly with regard to inhibition of the proliferation of precancerous and malignant lesions and/or to improvement of the effectiveness of conventional chemotherapeutic agents. NP technology is being widely used in prostate cancer, both in diagnostic and therapeutic fields, providing a promising strategy for drug delivery; such approaches with NP technology can make up for the lack of specificity of traditional therapy. Emerging NP technologies use for imaging and therapy in prostate cancer will be discussed in this section, with the aim of highlighting novel advances in NP technology for applications in prostate cancer.

### Imaging in Prostate Cancer

Magnetic resonance imaging and CT are two of the most critical and widely used methods for disease staging and diagnosis. In both methods, magnetic NPs (MNPs) can provide an encouraging solution as precursors for targeted contrast agents. MNPs in the form of superparamagnetic iron oxides (SPIOs) and ultrasmall SPIOs (USPIOs) have already been applied in clinical trials, with several advantages, including long blood-retention time, biocompatibility, and minimal biotoxicity (Feldman et al., 2008; Sun et al., 2008). Huang colleagues formulated new cationic lipid NPs containing SPIOs (L-SPIOs) to solve the problem of low efficiency of direct loading into cells and the cytotoxicity of SPIOs. The study used PC3 prostate cancer cells to test the efficiency of cellular uptake of these L-SPIOs, and the results showed that these L-SPIOs, which exhibited high loading efficiency, low cytotoxicity, and long-term imaging signals, are versatile image probes for cell tracking (Huang et al., 2009). Other metallic materials are also being developed. For example, Ghasemian et al. (2015) recently showed the potential applications of cobalt zinc ferrite NPs (CZF-MNPs) as a T2 contrast agent. *In vitro*, dimercaptosuccinic acid (DMSA)-coated or uncoated CZF-MNPs were incubated with PC3 or DU145 prostate cancer cells for 2 h at room temperature. Using a T2-weighted MRI system, researchers found that both uncoated and coated NPs were imported in PC3 and DU145 cells, resulting in a dramatic signal intensity reduction. In addition, the enhanced intensity observed using CZF-MNPs was similar to that of an equal amount of DMSA-coated CZF-MNPs, indicating that these particles were both suitable for T2 contrast enhancement (Ghasemian et al., 2015).

Photoacoustic imaging (PAI) is an emerging non-invasive imaging modality that combines the spectral selectivity of molecular excitation by laser light with the high resolution of ultrasound imaging. Some types of NPs, particularly gold NPs (GNPs), are designed for high photoacoustic contrast based on surface plasmon resonance (SPR) to enhance absorption (Agarwal et al., 2007; Yang et al., 2009). In addition to its apparent clinical safety and tolerability due to its physical inertness, gold can be used to synthesize NPs with very precise sizes, shapes, and surface chemistries at the nanoscale using simple techniques and relatively inexpensive reagents (Daniel and Astruc, 2004). A study demonstrated that PAI based on a targeted gold nanorod contrast agent could provide a clear anatomical view with targeted tissue highlighted in a contrasting color in LNCaP prostate cancer cells (Agarwal et al., 2007). The gold nanorods could be also used for detection of other lesions. Currently, GNPs are being used in several clinical trials as radiopaque markers that can be implanted into patients with prostate cancer for image-guided therapy (Kliton et al., 2014; Jorgo et al., 2017). The results showed that implantation had no apparent side effects, such as fever or infection, and none of the patients required analgesics after implantation, suggesting that GNPs could be potential imaging agents for use in clinic applications.

Molecular targets of prostate cancer, including prostate-specific antigen (PSA), prostate-specific membrane antigen (PSMA), and prostate cancer lipid antigen, are products of prostate cells and are used as potential NP targets (Cheng et al., 2012; Sanna and Sechi, 2012). Normal PSA immunoassays are often not able to detect PSA in the serum after radical prostatectomy due to the low sensitivity of the assays. However, using an extremely sensitive nanotechnology-based tool known as the “bio-barcode” system, the sensitivity of immunoassays has increased by 300-fold compared with that of commercial immunoassays, and the results of clinical studies have also confirmed these findings. This ultrasensitive technology is based on GNP probes decorated with DNA and antibodies that can recognize and bind to PSA when presenting at extremely low levels in blood samples (Thaxton et al., 2009). GNPs have also shown great efficiency and robustness as probes for sensing ultralow levels of PSA in patient serum samples. The limit of detection of the probe for PSA reached 32 fg/mL, which is more than two orders of magnitude lower than that of the conventional fluorescence probe and even lower than that of the GNP bio-barcode assay (330 fg/mL) (Liu et al., 2013). Many other nanomaterials, such as carbon nanotubes (Patra et al., 2015) and quantum dots (Brazhnik et al., 2015), which can detect PSA at low levels and do not harm the body or normal cells, are also being applied as sensors for detection of PSA.

### Therapeutics in Prostate Cancer

In the clinical setting, prostate cancer can roughly be classified as localized, expansively localized, or metastatic. Localized cancer is thought to be curable, whereas metastatic cancer leads to androgen-independent progression and death within a few years (Salvador-Morales et al., 2009). Traditional methods include chemotherapy using chemopreventive agents and radical prostatectomy, and new methods, such as cryosurgery, radiation with conformal external beam radiation, and brachytherapy, have also recently emerged. However, current treatments for prostate cancer are not sufficiently reliable for targeting of neoplastic cells without damaging normal cells. Finding new ways to overcome the disadvantages of conventional therapy methods is necessary, and nanotechnology may provide one such relevant approach. **Table 1** provides an overview of NPs that are promising for prostate cancer treatment.

**Table 1:** Overview of NPs designed for urological cancer treatments.

Cancer types	Treatment strategies	Applied NPs	Reference
Prostate cancer	Chemotherapy	GNPs loaded with chrysophanol	Lu et al., 2017
		EGCG-encapsulated polymeric NPs modified with PSMA	Sanna et al., 2011
	Gene therapy	Polymeric NPs loaded with GS25	Voruganti et al., 2015
		GNPs loaded with miRNA	Ekin et al., 2014
	Thermal therapy	MNPs and GNPs	Johannsen et al., 2007a, 2010
Renal cancer	Targeted therapy	Sorafenib-loaded PLGA NPs, DPPC liposomes and HMC-coated DPPC liposomes	Liu et al., 2015a,b
	Gene therapy	FA-PEAs:VHL complexes	Sudimack and Lee, 2000
	Thermal therapy	PEGylated gold nanoshells	Pannerec-Varna et al., 2013
		MWCNTs	Burke et al., 2009
	PDT	GNPs with radiofrequency radiation	Nikzad et al., 2017
	Targeted therapy	Cuprous oxide nanoparticles	Yang et al., 2017
		Superparamagnetic-like particles	Leulmi et al., 2015
Bladder cancer	Immunotherapy	Liposomes encapsulating BCG’s CWS	Nakamura et al., 2014
	Gene therapy	PEGylated cationic liposome carriers (PCat, PPCat) and modified PLGA NPs delivering siSurvivin	Martin et al., 2014; Cui et al., 2015
	Targeted therapy	PLGA NPs loaded with belinostat	Martin et al., 2013
	Chemotherapy	Paclitaxel-loaded protein NPs	Lu et al., 2011; McKiernan et al., 2011
	PTT	GNPs	Chen et al., 2015b
		Silica-Au nanoshells, Au/Ag nanospheres, and Au nanorods modified with tumor targeting antibodies	Juarranz et al., 2008; Cheng et al., 2009
		NP-AAG	Long et al., 2018


Many studies have shown that natural products are potent chemopreventive agents in prostate cancer due to their considerable efficacy against tumor cells and low toxicity (Jeong et al., 2011; Sanna et al., 2013; Kumar et al., 2015). For example, a recent study on chrysophanol, a natural compound extracted from plants of the genus *Rheum*, using GNPs as carriers to improve its bioavailability and water solubility successfully showed that chrysophanol suppresses human prostate cancer progression at the molecular level (Lu et al., 2017). Molecular targeted cancer therapy mediated by NPs is a promising strategy to overcome the non-specific biodistribution and therapeutic index of conventional chemotherapeutic drugs. Importantly, PSMA-modified NPs have been developed as a powerful treatment for prostate cancer. By conjugating PSMA to NPs, the functional particles can have good selectivity and target drugs, such as natural products or other chemotherapeutic drugs, to diseased tissue. For example, a natural product isolated from green tea, (–)-epigallocatechin 3-gallate (EGCG) shows potent chemopreventive effects in *in vitro* and *in vivo* models of prostate cancer, and several clinical trials have been conducted to determine the ability of green tea extracts to prevent the development and progression of prostate cancer (Stuart et al., 2006). A recent study showed that EGCG may be beneficial in the early stages of prostate cancer. Sanna colleagues designed biocompatible polymeric EGCG-encapsulated NPs modified with PSMA to selectively deliver EGCG to prostate cancer cells. Experiments *in vitro* demonstrated that these novel NPs could lead to increased antiproliferative activity toward PSMA-positive prostate cancer cells, without affecting normal cell viability (Sanna et al., 2011). Similarly, by conjugating GNPs with an ONT-hybridized, PSMA-specific aptamer, a GC-rich duplex acting as a loading site for doxorubicin was formed. Kim et al. (2010) established multifunctional NPs for both prostate imaging and treatment. This novel NP bioconjugate not only enabled visualization of target-specific binding by silver staining and clinical CT scanning but also efficiently induced cell death in prostate cancer cells (Kim et al., 2010).

Gene therapy is an extensive method used for the treatment of prostate cancer. Generally, there are four main strategies for gene therapy: tumor-suppressor therapy, suicide gene therapy, immunomodulatory gene therapy, and anti-oncogene therapy. However, none of these approaches can be applied alone as therapeutic treatment due to the low specificity of cell targeting and inefficient gene transfer and expression (Djavan and Nasu, 2001). NPs are widely used in anti-oncogene therapy to overcome these problems, and many studies have used nanodelivery systems to enhance the activities of oncogene inhibitors. For example, 25-OCH_3_-PPD (GS25) is a natural inhibitor of the *MDM2* oncogene, which can be amplified and/or overexpressed in prostate cancer. When loaded with polyethylene glycol-poly(lactic-co-glycolic acid) NPs, GS25 showed enhanced anticancer efficacy *in vitro* and *in vivo* without inducing toxicity and exhibited improved uptake in cancer cells (Voruganti et al., 2015). MicroRNAs are commonly used as tools in gene cloning to induce post-transcriptional gene silencing. Some studies have shown that GNP-based nanocarriers (Ekin et al., 2014) and prostate cancer-targeted polyarginine-disulfide linked polyetherimide (PEI) nanocarriers (Zhang et al., 2015) can promote *miR-145* delivery into prostate tumors.

Magnetic fluid hyperthermia (MFH) is another promising method of prostate cancer treatment using biocompatible MNPs injected directly into superficial or deep-seated tumors and consecutively heated in an alternating magnetic field (Johannsen et al., 2010). Because tumor cells have a lower heat tolerance than normal cells, increasing the temperature to 40–43°C by hyperthermia (HT) can lead to tumor cell necrosis and apoptosis. MNPs play an important role in this novel therapy due to the excellent power absorption capabilities of magnetic fluids in a magnetic field (Jordan et al., 1993). A prospective clinical study investigated MFH as a monotherapy used in patients with locally recurrent prostate cancer and showed that this approach was feasible and well-tolerated; additionally, deposition of NPs in the prostate was highly durable (Johannsen et al., 2005a, 2007a,b). Recent studies have shown that HT combined with radiotherapy (RT) may be effective for the treatment of prostate cancer (Hurwitz et al., 2011; Datta et al., 2015), and MFH can effectively enhance RT, leading to significant growth inhibition in mouse models of human prostate cancer (Johannsen et al., 2005b; Attaluri et al., 2015).

In addition to MNPs, GNPs have also shown several advantages in HT therapy, including good biocompatibility. In a study of the influence of different physical characteristics (shape, size, surface properties, and concentration) of GNPs on cellular uptake, adsorption of proteins, and toxicity in PC3 human prostate cancer cells, size-dependent uptake and negligible toxicity were observed (Arnida et al., 2010). Similar to HT therapy, thermal ablative energy causes necrosis of target tissues by inducing a high temperature, leading to focal cell death and preserving the circumambient normal tissue (Sanna et al., 2011). Studies using systemically administered non-targeted NIR-activatable gold nanoshells for thermal ablation of tumors date back to the early 21st century; however, very few reports have documented its utility in the treatment of prostate cancer (Krishnan et al., 2010). Stern et al. (2007) studied gold nanoshell-mediated photothermal ablation of prostate cancer cells and found complete loss of cell viability while maintaining intact cellular morphology. A subsequent study evaluated the efficacy of laser-activated gold nanoshell thermal ablation for eradicating prostate cancer *in vivo* and compared the therapeutic efficacies of two doses of gold nanoshells (7 and 8.5 μL/g body weight). The results demonstrated that the low concentration of gold nanoshells arrested cell growth, whereas the high concentration of gold nanoshells dramatically decreased tumor volume (93% tumor necrosis and regression) within just 1 week (Stern et al., 2008). Other applications of GNPs are also being investigated. For example, genetically modified phages (with both gold-binding peptide and PC3-binding peptide) were induced to attract GNPs to form a cluster, enabling stable maintenance of cell-targeting functionality and killing of the targeted prostate cancer cells within a short time. The prostate cancer cells were killed more efficiently and selectively than non-GNP-treated cells (Oh et al., 2015).

## Nanotechnology in Renal Cancer

Renal cell carcinoma (RCC) is not a single entity, but includes a group of tumors with a highly heterogeneous epithelium originating from the renal tubules. There are three dominant histopathological types of RCC: clear cell (65%), papillary (15–20%), and chromophobe (5%) (Bouchelouche, 2016; Yuan et al., 2016). RCC is the third most common urinary system cancer, with an incidence rate of about 5–10 cases per 100,000 people, and accounts for 2–3% of all malignant tumors (Xu et al., 2010). Although chemotherapy is one of the main modes of cancer treatment, its effectiveness is limited by drug resistance. For example, sunitinib is currently the standard first-line treatment for advanced RCC, and patient inevitably become resistant to sunitinib treatment (Yang et al., 2017). In addition, the clinical outcomes in patients with RCC remain poor. Therefore, rapid development of nanotechnologies may improve therapeutic strategies for the diagnosis and treatment of RCC (Yang et al., 2017).

### Imaging in Renal Cancer

Pinpoint imaging of RCC is one of the main issues for the diagnosis, staging, and clinical treatment of patients (Bouchelouche, 2016). In current oncology, a series of NPs for diagnostic assays have been developed, and these NPs are commonly used as contrast agents for MRI and CT imaging (Sanna et al., 2011; Williams et al., 2016).

Lu et al. (2014) conjugated the monoclonal antibody G250 and SPIO into molecular MRI probes for the *in vitro* detection of clear cell RCC (ccRCC) using a 3.0-Tesla MRI. An *in vitro* MRI study of ccRCC and control cells confirmed that the fabricated mAb G250-SPIO nanoprobe could be used as a specific marker for ccRCC cells (Lu et al., 2014). Moreover, the nanoprobe could be easily conjugated with other antitumor drugs and imaging materials to achieve multimodal imaging and diagnostic treatment integration. For example, the combination of Cy5-labeled glycosylated bleomycin (Yu et al., 2013; Bhattacharya et al., 2014) and the nanoprobe may provide a new direction for the design of novel tumor imaging and therapeutic agents. Besides, GNPs or quantum dots as imaging materials (Mieszawska et al., 2013; Zrazhevskiy and Gao, 2013) could also be developed with the mAb G250 nanoprobe to promote highly multiplexed parallel staining, resulting preferable imaging.

Many studies have demonstrated that the diagnosis of RCC can be performed by multifarious NP imaging. However, urologists are often faced with imaging dilemmas, including lack of definitive answers in clinical practice during the diagnosis of RCC histology (Farber et al., 2015). In addition, whether contrast-enhanced imaging of RCC has any side effects in patients is worth considering. We expect to see a growing number of nanotechnologies to fill technological gaps in the diagnosis and treatments of RCC.

### Therapeutics in Renal Cancer

Nanomedicine strategies may be more effective than other strategies for controlling drug delivery and release. Due to the high porosity of the tumor vasculature, impaired lymphatic drainage, known as the enhanced permeability and retention effect, or the high affinity of the NPs for markers specifically expressed on cancer cells can result in easy accumulation at the tumor site. Therapeutic nanotechnology focuses on the utilization of photothermal-based ablative energy, which is sent to the targeted lesion by activating various types of NPs (Sanna et al., 2011). Typical nanomedicine delivery vehicles include liposomes, polymeric NPs, nanoshells, and MNPs (**Table 1**).

Many preclinical studies have examined liposomal strategies for drug delivery in RCC; however, only a few clinical studies have been conducted (Williams et al., 2016). Polyethylene glycol (PEG)-ylated-liposomal doxorubicin (Doxil) was used for the treatment of patients with intractable RCC in a phase II clinical trial. The results showed that Doxil was ineffective in patients with RCC, although slightly toxic side effects were observed (Skubitz, 2002). Liu et al. (2015a,b) systemically estimated the *in vitro* applicabilities of several drugs as delivery systems for the treatment of RCC, including sorafenib-loaded poly(lactic-co-glycolic acid) (PLGA) NPs, 1, 2-dipalmitoyl-sn-glycero-3-phosphocholine (DPPC) liposomes, and hydrophobically modified chitosan (HMC)-coated DPPC liposomes. The results showed that sorafenib-loaded PLGA NPs and HMC-coated DPPC liposomes could apparently kill more RCC cells than sorafenib alone at lower concentrations. Moreover, XL184 liposomes counteracted tumor activity by inhibiting the phosphorylation of Met, AKT, and mitogen-activated protein kinase pathways in RCC cells (Kulkarni et al., 2016). The above studies indicated that liposomes may have applications in multikinase pathway inhibition as a prospective treatment for RCC.

Folic acid-modified poly(ε-caprolactone)-pluronic-poly(ε-caprolactone) grafted isophorone diidocyanate-PEI (FA-PEAs) was developed as a low-toxicity carrier to transfer Von Hippel-Lindau (VHL) plasmids to treat mice in an RCC model. Johnson et al. (2010) found that the mean tumor volume in FA-PEAs:VHL-treated mice was decreased about 30% compared with that in the control group. Due to the limited expression of folate receptors on healthy cells and overexpression in RCC, FA-PEAs:pVHL complexes could specifically bind to the cell surface via a ligand–receptor incorporating mechanism (Sudimack and Lee, 2000). Thus, the FA-PEAs:VHL complexes could be released into the cytosol through endocytosis to exert therapeutic effects.

Currently, sunitinib is the standard first-line drug for RCC; however, patients inevitably develop resistance to this drug, leading to therapy failure and poor prognosis (Joosten et al., 2015; Stone, 2016). Therefore, new therapies are needed to improve treatment outcomes in patients with RCC. The rapid development of nanotechnology has provided emerging techniques for the treatment of drug-resistant RCC. Yang colleagues demonstrated that cuprous oxide NPs can markedly inhibit RCC tumor growth with minimum renal toxicity *in vitro* and *in vivo*, thereby reversing sunitinib resistance. The mechanism may involve the downregulation of copper chaperone proteins antioxidant 1 copper chaperone and copper chaperone for superoxide dismutase in RCC cells, influencing copper trafficking to the endoplasmic reticulum and mitochondria and thereby initiating endoplasmic reticulum stress and mitochondrion-mediated apoptosis by activating caspase-3, caspase-9, and caspase-12 (Yang et al., 2017). In addition, cell cycle and apoptosis regulator 1 functional mimetic compounds (CFMs) inhibit cell growth by inducing apoptosis in various cancer types, even drug-resistant cancer (Muthu et al., 2015). Cheriyan et al. (2017) found that CFM-4.16 had strong inhibitory effects on parental and everolimus-resistant RCC cells. Because of the poor aqueous solubility of CFM-4.16, it was encapsulated in PEGylated vitamin E-based nanomicelles generating water-soluble formulations with higher drug loading (30% w/w). The findings of this study suggested that CFM-4.16 nanomicelles inhibited the viability of parental and everolimus-resistant RCC cells *in vitro* and suppressed the growth of RCC cell-derived xenografts (Cheriyan et al., 2017).

The use of PEG-conjugated antibodies attached to nanoshells guarantees biocompatibility for drug delivery to tumor cells. Pannerec-Varna et al. (2013) developed large PEGylated gold nanoshells with distribution in the different cellular components of human renal cancer. The distribution kinetics progressed from intravascular flow at 30 min to intratumoral cells 24 h later, and no toxicity was detected in mice with PEGylated gold nanoshells at 6 months. This novel research provided important insights into the use of large PEGylated gold nanoshells by optimizing the time of targeted hyperthermia or topical drug delivery to carcinoma cells (Pannerec-Varna et al., 2013). Additionally, new studies have shown that diverse tumor types can be evaluated preclinically and clinically using CRLX101, an NP-drug conjugate containing camptothecin conjugated to a biocompatible cyclodextrin/PEG copolymer (Gaur et al., 2014; Lin C.J. et al., 2016). CRLX101 in combination with bevacizumab has been assessed in a phase I trial of mRCC treatment (NCT01625936^1^), and the initial data indicated that CRLX101 combined with bevacizumab was an adequate therapy in mRCC. The strategy of using biodegradable polymer NPs to control the delivery of medicines for multiple types of cancers has become feasible, although the therapeutic effects of these biodegradable polymer NPs in RCC are unknown (Schroeder et al., 2012; Devulapally and Paulmurugan, 2014). Nevertheless, Abraxane (albumin NPs conjugated to paclitaxel) was well-tolerated for the treatment of metastatic urothelial carcinoma and showed tumor responses in 27.7% of patients, revealing that this strategy may be a valid second-line treatment (Ko et al., 2013).

Gold NPs can be used in cancer treatment due to their remarkable compatibility, tunable stability, and low toxicity. Moreover, GNPs are often used in photodynamic therapy (PDT) by generating heat to kill tumor cells (Cabuzu et al., 2015). Nikzad et al. (2017) assessed the effects of treatment of RCC with RF radiation in the presence of GNPs. Human embryonic kidney cells were subjected to various tests in the presence of RF and GNPs. *In vitro* MTT assays showed that cell viability was dramatically reduced in the RF-treated groups in the presence of GNPs compared with that in the control group. Thus, this method may be suitable for the treatment of RCC, providing a new method for alternative nephrectomy (Nikzad et al., 2017).

Sreekanth et al. (2016) used sulforhodamine B assays and two-color flow cytometry analysis to test the toxic effects of CdO nanostructures on Madin-Darby canine kidney epithelial cells (MDCK cells) and Caki-2 human renal cancer cells. Compared with normal cells, CdO had significant cytotoxic effects on MDCK and Caki-2 cells. Thus, CdO could inhibit the growth of tumor cells and may be an effective drug for the treatment of RCC (Sreekanth et al., 2016).

**FIGURE 2:**
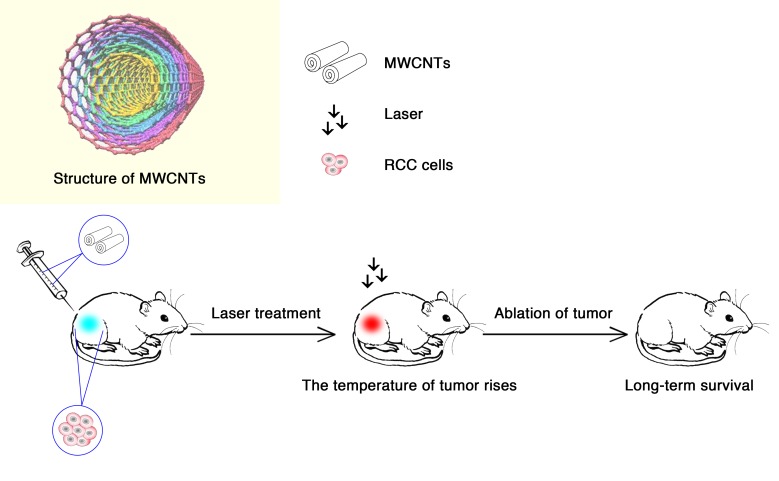
Multiwalled carbon nanotubes (MWCNTs) ablate tumors. Tumor-bearing mice were injected intratumorally with 50 μL of treatment solution containing 100, 50, or 10 μg of MWCNTs (Burke et al., 2009). Tweety-four hours after the injection, they were irradiated with a 1,064-nm NIR laser for 30 s (3 W/cm^2^; spot size, 5 mm). In the presence of the laser, MWCNTs increased intratumoral temperatures to a level sufficient to induce tumor ablation, resulting the long-term survival in tumor-bearing mice.

Burke et al. (2009) confirmed the heating effects of treatment with multiwalled carbon nanotubes (MWCNTs) through magnetic resonance temperature-mapping and heat shock protein-reactive immunohistochemistry. The results showed that the use of MWCNTs could ablate tumors with low laser power and reduce treatment times, with minimal local toxicity and no significant systemic toxicity (**Figure 2**). MWCNTs are thermal ablation agents that can lead to long-term survival in tumor-bearing mice, which indicates that the combination of MWCNTs and laser may be a promising method for the treatment of RCC (Burke et al., 2009).

Recently, because of the low frequency of mechanical effects on cancer cell membranes, some original approaches have been developed to eradicate cancer cells. A study on human renal cancer cells with superparamagnetic-like particles was conducted by Leulmi et al. (2015), who confirmed that apoptosis could be achieved by the vibration of MNPs attached to cancer cells. With regard to the invasiveness of the treatment, magneto-mechanical stimulation is superior to magnetic hyperthermia and is easy to put into practice (Drexler, 1986).

## Nanotechnology in Bladder Cancer

Bladder cancer is the seventh most commonly diagnosed cancer in the male population worldwide (Babjuk et al., 2017), and can be divided into non-muscle-invasive bladder cancer (NMIBC) and muscle-invasive bladder cancer (MIBC) according to the pathologic stage (Jiang et al., 2015). At diagnosis, approximately 75% of patients with BCa present with NMIBC (Babjuk et al., 2017). The most common method for detection of NMIBC is standard white light cystoscopy; however, it is difficult to detect flat lesions and differentiate benign from malignant lesions. TUR-BT, as the gold standard for primary treatment for NMIBC, frequently leads to tumor relapse. To prevent recurrence, various medicines, including BCG and mitomycin C, have been used extensively through intravesical instillation as adjuvant therapy after TUR-BT. However, these treatments can cause septicemia, disseminated intravascular coagulation, and multiple organ failure because of severe toxicity (Lodde et al., 2014). Therefore, more advanced diagnosis and therapies are needed (**Table 1**).

### Imaging in Bladder Cancer

For the detection of NMIBC, fluorescent or photodynamic diagnosis has been developed as an improvement for contemporary white light microscopy. Photosensitizers, such as hexaminolevulinate, are used in fluorescence cystoscopy and are preferentially absorbed by cancer cells, resulting in a red appearance in comparison with surrounding tissues. Although these compounds exhibit high sensitivity, they may show low specificity, particularly after BCG therapy (Ray et al., 2010). Previous studies have shown that nanotechnology can enhance the specificity of photosensitizers to tumor cells and improve the identification of cancer by increasing the uptake of NPs within tumor cells (Olivo et al., 2012; Yan et al., 2013). Recently, Lin T.Y. et al. (2016) developed a multifunctional nanoporphyrin called PLZ4-nanoporphyrin (PNP) due to its coating with the BCa-specific ligand PLZ4. PNP displays the ability to preferably emit fluorescence/heat/reactive oxygen species upon illumination with near infrared light, allowing the fluorescence signal of PNPs to efficiently and selectively increase in BCa cells.

As metal NPs, GNPs can be modified with functional groups by being combined with ligands, antibodies, and other medicines (Cheng et al., 2014). Due to its characteristic SPR signal according to the distance of NPs, GNPs can modify protein molecules and particular nucleic acids to show different colors (Larguinho and Baptista, 2012). Because of SPR, colloidal GNPs are red in color and can be 4–5 orders of magnitude larger than the traditional dye in absorption and scattering cross-sections (Azzazy et al., 2006). These properties can be used to develop easier and faster methods for diagnosing diseases. When GNPs aggregate, their color changes from red to blue due to a second phenomenon known as plasmon-plasmon transfer (Huang and El-Sayed, 2010). The rationale is that GNPs are stabilized by the adsorption of single-stranded DNA through their nitrogenous bases, preventing the salt-induced aggregation of GNPs and keeping the solution red in color. In contrast, the repulsion between the GNP surface and double-stranded DNA (dsDNA) prevents dsDNA from adsorbing to the NP surface (Li and Rothberg, 2004, 2005); because of this, GNPs are not protected from salt-induced aggregation, and the color of the solution turns blue.

**FIGURE 3:**
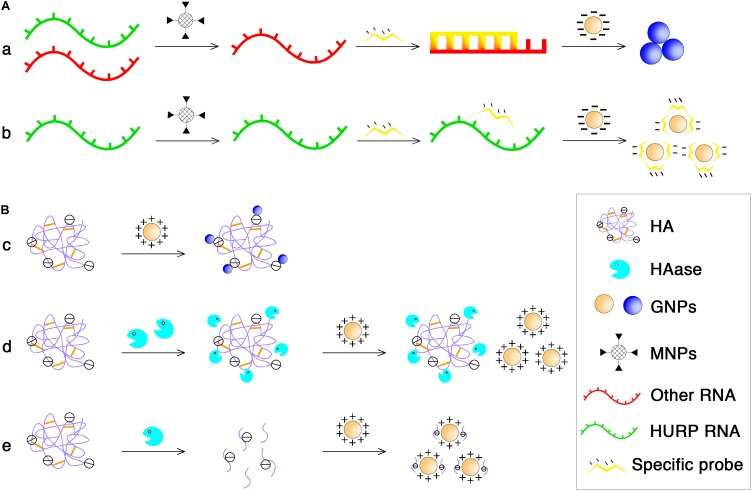
The principle of NP-based diagnosis of BCa. **(A)** MNPs and GNPs used in purifying total RNA to obtain HURP RNA. **(a)** MNPs functionalized with oligonucleotide sequences specific for HURP RNA were added followed by mixing with hybridization buffer which contained NaCl, and HURP specific oligonucleotide with purified HURP RNA. After denaturation and annealing, the negatively charged GNPs were added and in the presence of HURP RNA, hybridization occured between the oligotargeter and the HURP RNA, and the solution color shifted from red to blue due to salt-induced aggregation of GNPs. **(b)** Without HURP RNA, GNPs would not aggregate and the solution color remained red because the oligotargeter remained adsorbed onto the surface of the GNPs. **(B)** Cationic GNPs directly detected HAase activity. **(c)** The charge interaction between polyanionic HA and cationic GNPs can lead to the formation of gold aggregates and at the same time a red to blue color shifted. **(d)** Under conditions of high HAase concentration, the enzyme and HA binds electrostatically even at a 0-min incubation time at 37°C, no aggregation occurs when the GNP solution is added, and the solution remains red in color. **(e)** At low concentration conditions, enzymatical reaction at 37°C for a period of time could lead to the enzyme molecule digest HA into small fragments, resulting in the failure of GNPs aggregation.

Based on these phenomena, Eissa et al. (2014) have developed colorimetric GNP assays to detect unamplified hepatoma upregulated protein RNA (HURP RNA) in urine directly for BCa diagnosis. The principle is shown in **Figure 3A**. Briefly, the purified HURP RNA was mixed with hybridization buffer containing NaCl and a specific oligotargeter. After denaturation and annealing, hybridization between the oligotargeter and the HURP RNA occurred, and the added GNPs were aggregated without the protection of the oligotargeter. The solution color then shifted from red to blue. If there was no HURP RNA in the samples, the GNP surface was fully adsorbed with the oligotargeter, and the solution color remained red. Using this sensitive and non-invasive assay, BCa could be detected at an early stage, making it possible to begin treatment earlier and achieve better therapeutic effects. In another study, GNPs were also developed for rapid and sensitive detection of urinary hyaluronidase (HAase) activity (Nossier et al., 2014). The charge interaction between polyanionic hyaluronic acid (HA) and cationic GNPs stabilized with cetyl trimethyl ammonium bromide can lead to the formation of gold aggregates and a red to blue color shift. After the enzymatic reaction of HAase, the polymeric HA is degraded into small fragments that cannot aggregate GNPs (**Figure 3B**). Similarly, the developed cationic GNP assay was used for qualitative detection of urinary HAase activity (a urinary marker of BCa) in patients with bladder carcinoma by detecting the absorption ratio (*A*_530_/*A*_620_) of the reacted GNP solution, which showed a better sensitivity (82.5%) than that of zymography (Nossier et al., 2014).

Nuclear matrix protein 22 (NMP22) is a monitoring tool for predicting the recurrence/clearance of BCa and for screening undiagnosed individuals who have symptoms or are at risk for the disease. Lee colleagues demonstrated a novel strategy to sense NMP22 in urine samples by using a sensing element comprised of molecularly imprinted polymers (MIPs) to specifically recognize NMP22 molecules. In this study, zinc oxide nanorods were hydrothermally grown on the sensing electrodes to increase the surface area to be coated with MIPs and therefore enhance the electrochemical response (Lee et al., 2016).

### Therapeutics in Bladder Cancer

Liposomes as carriers of molecules, such as small-molecule cytotoxic agents (Guhasarkar et al., 2017), nucleotides (Cuomo et al., 2013), and proteins (Vila-Caballer et al., 2016), have also been developed for BCa therapy. To improve the poor solubility and enhance the efficiency of the cell wall skeleton (CWS) of BCG, Nakamura’s group encapsulated the CWS of BCG within liposomes (Nakamura et al., 2014). Recently, they modified cationic liposomal surfaces with cholesteryl-PEG to overcome urine aggregation and promote cellular uptake into cancerous urothelial tissues (Nakamura et al., 2017). Liposomes can be also used to envelop small interfering RNA (siRNA) silencing oncogenes or other tumor-related genes for the prevention or treatment of BCa. Cui et al. (2015) adopted two newly developed PEGylated cationic liposome carriers (PCat, PPCat) for delivering survivin siRNA (siSurvivin) and enhancing the activity of mitomycin C in human BCa cells. A fusogenic lipid was used in both carriers to destabilize the endosomal membrane, and PPCat further contained paclitaxel to enhance the *in vivo* delivery and transfection of siSurvivin.

Several studies have shown that polymeric NPs as drug carriers have great promise in the treatment of BCa (Huang et al., 2012; Yan et al., 2013). To utilize polymeric NPs as carriers, the polymers must have a biodegradable structure and show changes in composition to carry a specific drug to a specified location to achieve targeted drug delivery. The improved polymeric NPs also need to be less expensive, easier to synthesize, and non-immunogenic. One of the most widely used polymeric NPs is PLGA NPs, which show outstanding biocompatibility, biodegradability, ability to be modified with polymers and peptides (Cu et al., 2011), and ability to facilitate controlled release of loaded drugs (Hua et al., 2014). Martin et al. (2013) designed PLGA NPs loaded with belinostat, a histone deacetylase inhibitor, for BCa treatment. PLGA NPs were surface modified with a novel cell penetrating polymer, poly(guanidinium oxanorbornene). The results showed that intracellular uptake was also enhanced significantly and tissue penetration ability was improved by 10-fold in intravesically treated mouse bladders and *ex vivo* human ureters. In a subsequent study, researchers used a low-molecular-weight (2.5 or 20 kDa) positively charged mucoadhesive polysaccharide, chitosan, to modify the surface of PLGA NPs in order to deliver siSurvivin to the BCa cells (Martin et al., 2014). These surface-modified PLGA NPs could not only cross the urothelium and target the exact tumor site but also load a large number of siRNAs.

Protein NPs have been used as drug carriers in the treatment of many cancers (Lee et al., 2017; Palchetti et al., 2017). Using proteins as carrier materials *in vivo* may solve enzyme-induced degradation problems, reduce the effects of drug toxicity, and offer good biocompatibility compared with other types of materials, such as metals. The production of urine during treatment leads to dilution of the drug concentration used for intravesical instillation, resulting in the failure of intravesical treatment of NMIBC. However, studies have found that the use of paclitaxel-gelatin NPs can solve the problem of drug dilution by newly produced urine through constant drug release and that achieving a constant drug concentration can reduce the frequency of treatment. For example, paclitaxel-loaded gelatin protein NPs have been developed to improve the delivery and retention properties of paclitaxel in intravesical therapy against BCa (Lu et al., 2011). Compared with docetaxel, NP albumin-bound paclitaxel (Nab-paclitaxel) has been shown to have increased solubility and lower toxicity in systemic treatment, and minimal toxicity and systemic absorption were demonstrated in the first human intravesical phase I trial (McKiernan et al., 2011). Phase II trials have shown that Nab-paclitaxel is well-tolerated in patients with pretreated advanced urothelial cancer and has a good tumor response (Ko et al., 2013). These results have highlighted the potential applications of Nab-paclitaxel in cancer treatment.

Recently, novel MNPs coated with three broad-spectrum lectins, namely concanavalin A (ConA; MNP@ConA), wheat germ agglutinin (WGA; MNP@WGA), and Sambucus nigra (SNA; MNP@SNA), have been developed to selectively capture glycoproteins from the urine of patients with different types of BCa (Azevedo et al., 2018). In this case, lectins were used for targeting glycan moieties in cancer-associated glycoproteins, indicating the possibility of targeting local release of drugs.

The optical properties of SPR are used to assist laser-induced thermotherapy in plasmonic photothermal therapy (PTT) with GNPs to enhance the effects of target therapy (Nergiz et al., 2014), avoiding damage to neighboring normal tissues because of the hyperthermic state induced by the relatively low laser power. Chen et al. (2015b) showed that GNPs require only half the laser power of regular laser treatment, and Cheng et al. (2009) demonstrated that silica-Au nanoshells, Au/Ag nanospheres, and Au nanorods modified with tumor targeting antibodies, such as anti-epidermal growth factor receptor and anti-epidermal growth factor receptor 2 antibodies, were internalized by tumor cells, resulting in photothermal-induced death of BCa cells *in vitro*. Among the three types of nanomaterials, silica@Au nanoshells require a minimum number of particles to produce effective photodestruction. In contrast, PDT is also a promising strategy for BCa therapy, involving the use of photosensitizers, which are usually large cyclic compounds, such as porphyrins, chlorins, and phthalocyanines. These substances will produce free radicals when exposed to near infrared or visible light. However, the products currently on the market have quite a few limitations, including specific differences in the ability to act on the tumor alone (Juarranz et al., 2008). In a recent study, Long et al. (2018) designed a novel multifunctional nanoporphyrin platform loaded with the heat-shock protein 90 inhibitor 17AAG (NP-AAG) and found that bladder tumors could be synergistically eradicated with a single intravenous injection of NP-AAG followed by multiple light treatments within 7 days.

## Nanotechnology in Other Urological Cancer

Other urological carcinomas have received limited attention with regard to nanotechnology applications. Feldman et al. (2008) conducted a preliminary study of the potential of USPIOs for accurate detection of lymph node metastases of penile and testicular cancers through lymphotropic NP-enhanced MRI. Patients with penile carcinoma underwent a pelvic MRI after using USPIOs. The results showed that better sensitivity, specificity, and accuracy could be obtained by comparing conventional imaging techniques, suggesting that the pathological status of local lymph nodes could be accurately predicted by MRI in patients with penile cancer (Tabatabaei et al., 2005; Mueller-Lisse et al., 2008; Hughes et al., 2009). Similarly, a prospective pilot study examined the use of lymphotropic NP-enhanced MRI to detect lymph node metastasis in patients with testicular cancer after injection of USPIOs. The data suggested that USPIOs greatly improved the specificity and sensitivity of accurate lymph node metastasis diagnosis (Harisinghani et al., 2005).

## Future Perspectives

Despite the use of nanotechnologies with tremendous potential for the detection, diagnosis, and treatment of urological cancers, more studies are still required. First, safety profiles must be studied in detail because of insufficient knowledge regarding the biocompatibility of nanocarriers. The safety of GNPs and gold nanoshells has been widely evaluated in preclinical trials, and full clinical trials are still underway for many of these materials. USPIOs have been under continued scrutiny, and ferumoxtran-10 is currently entering phase III clinical trials in Europe (Daldrup-Link, 2017). Additionally, iron-oxide NPs have been approved as an MRI contrast agent by the US Food and Drug Administration (Wang et al., 2014). A database on health risks linked to different types of NPs should also be established according to different pharmacokinetic behaviors. Second, additional research into the design and development of NPs with multiple functions (single or dual imaging, targeted therapy, and combination therapy) is needed. Moreover, the development of multifunctional NPs loaded with targeted agents may eventually be permitted for both PTT and targeted therapy to achieve simultaneous detection and treatment of urological cancer. Third, a widely used and well-accepted dosimetry model is needed to estimate the NP concentration in tumors of interest. Dosimetry formulations have been used to guide radiation therapy, but not for PTT because of the uneven distribution of particles and the effects of tissue physiology. Thus, further studies are required to establish such formulas. Lastly, in the future, more studies are needed to examine the environmental influence of manufactured NPs, which are deliberately produced with specific properties; these NPs have been shown to enter air, water, and soil from a range of routes (Kumar et al., 2012). Thus, there is an urgent need for the development of safety guidelines for NPs.

## Conclusion

During recent decades, many efforts have been made to combine novel technologies and conventional methods to combat cancer. The potential applications of nanotechnology were reviewed in this article. Nanotechnologies are promising tools to enhance the specificity and sensitivity of both the diagnosis and treatment of cancer and have been shown to have several feasible applications in urological cancer. Many studies and clinical trials have demonstrated that NPs can overcome the limitations of current medical approaches. Various metallic nanomaterials are used as contrast agents for MRI and CT imaging, providing a higher probability of detecting early urological cancer. The excellent photothermal properties of GNPs have led to their extensive application in imaging. Moreover, by application in HT therapy and thermal therapy, GNPs have shown potential to generate heat more efficiently. In contrast, many researchers have clearly demonstrated that NPs are ideal candidates for drug delivery systems because of their special size and are able to combine the benefits of drug delivery systems with improved safety and efficacy. Polymeric NPs have the capacity to be used for various applications from imaging to drug delivery systems. Several different types of polymeric materials can be used, and well-developed fabrication techniques have enabled them to be mass produced. Multifunctional NPs have many advantages because they are uniquely designed to target specific cancer cells. However, there are still some issues that need to be clarified, such as the safety of these NPs when used in the human body. Additional studies are still needed to clarify the details of nanotechnology-based methods and to establish these NPs as tools for the treatment of cancers.

## Author Contributions

M-HH, LC, TZ, and YT summarized the literatures and wrote the manuscript. QH and H-LF revised and edited the manuscript. J-CL, WZ, and GS provided critical comments and revised the manuscript. LH and Z-XY revised the manuscript and supervised all the works.

## Conflict of Interest Statement

The authors declare that the research was conducted in the absence of any commercial or financial relationships that could be construed as a potential conflict of interest.

## Footnotes

1 https://clinicaltrials.gov/
